# α- and β-Substituted Metal-Free Phthalocyanines: Synthesis, Photophysical and Electrochemical Properties

**DOI:** 10.3390/molecules25020363

**Published:** 2020-01-16

**Authors:** Hande Pekbelgin Karaoğlu, Ayfer Kalkan Burat

**Affiliations:** Chemistry Department, Technical University of Istanbul, İstanbul TR34469, Turkey

**Keywords:** fluorescence, hexadeca substitution, phthalocyanine, photophysics, synthesis, electrochemistry

## Abstract

Two novel phthalonitrile derivatives, bearing two hexyloxy groups and a benzodioxin (or a naphthodioxin) annulated ring, along with their corresponding metal-free phthalocyanines (H_2_Pc) were prepared. FT-IR, mass, electronic absorption, ^1^H NMR, and ^13^C NMR spectroscopy were employed for the characterization of all compounds. The effect of hexadeca substituents on the photophysical properties of metal-free Pcs was investigated. Photophysical properties of H_2_Pc were studied in tetrahydrofuran (THF). Fluorescent quantum yields of phthalocyanines (Pcs) were calculated and compared with the unsubstituted phthalocyanine. 1,4-Benzoquinone effectively quenched the fluorescence of these compounds in THF. Cyclic and square wave voltammetry methods were applied to metal-free phthalocyanines and Pc-centered oxidation and reduction processes were obtained.

## 1. Introduction

Phthalocyanines were first synthesized accidentally, and since then, they have been used as powerful artificial dyes and pigments in the paper and textile industries. This is because their thermal, chemical, and photochemical properties are excellent [1,2,3,4]. The redox chemistry of conventional Pcs is quite rich and they also have a delocalized 18 π-electron system; they also feature intense absorptions in near-IR. The coordination chemistry of Pcs encompasses the majority of elements of the periodic table, except for the most electronegative and radioactive elements. Organic semiconductors [5,6,7,8], photodynamic therapy (PDT) [9], catalysis [10], nonlinear optical devices [11], and chemical sensors [12] are typical industrial and medical applications of Pcs.

Functionalization of Pcs with suitable substituents improves their solubility, optimizes their photophysical and photochemical properties, and enables their use in specific areas. The insertion of the substituents at both peripheral (β-positions) and non-peripheral (α-) positions of the Pcs results in the formation of hexadeca-substituted Pcs that are comparatively less studied than the corresponding octa- and tetra-substituted derivatives. Studies about Pcs with different substituents at α- and β-positions have also been published [13,14,15,16,17]. Most of these studies involved the synthesis and characterization of novel hexadeca-substituted Pcs. In another study reported by Machacek et al., the anticancer activities of hexadeca-substituted cationic Pcs were investigated [17]. It is known that hexadeca substitution increases the solubility of the macrocycle by reducing aggregation [8,18,19,20,21]. In these studies, long alkyl chains, such as butoxy or hexyloxy, are attached to the non-peripheral positions of phthalocyanines to improve their solubility. Furthermore, if α-positions of Pcs were substituted with alkoxy substituents, a red-shifted Q band from 650 nm to 750 nm was observed, reducing the highest occupied molecular orbital/lowest unoccupied molecular orbital (HOMO-LUMO) gap [7,19,22,23,24,25,26,27,28]. With respect to Kobayashi’s papers from 2011 and 2014, non-peripherally S-, Se-, or Te-substituted Pcs exhibited Q bands beyond 1000 nm [29,30]. In another work, Gorbunova and coworkers reported a new series of molecules with a possible change of absorption in the near-infrared (NIR) region (687–1028 nm) [19]. These Pcs not only show strong bathochromic shifts but they also feature solvatochromic behavior resulting from J-aggregation via coordination between α-O atom and zinc ion of a neighboring molecule or protonation of the meso positions [31,32]. It is clear that the substitution pattern of Pc significantly influences the intermolecular π–π stacking and electronic coupling. In particular, hexadeca-substituted Pcs with different substituents are useful compounds for high-tech applications because of their red-shifted absorptions, high solubility, non-aggregation behavior, and existence in one positional isomeric form [33].

Hence, we focused on the preparation and investigation of Pcs carrying hexadeca substituents. In our previous work, π-extended hexadeca-substituted cobalt Pc (CoPc) was reported to evaluate its potential application as an organic field-effect transistor (OFET) [7]. The relatively high charge carrier mobility of CoPc indicates that hexadeca-substituted phthalocyanines can be used as active layers in OFETs. In another work of ours, synthesis, characterization, and electrochemical properties of biphenyl- and diphenylacetylenyl-based hexadeca-substituted metal-free Pcs were presented in detail, and differences in the HOMO energy levels for metal-free Pcs were obtained [28]. To examine the change in properties induced by the structural variation of appended groups to Pcs, we report here on benzodioxin- or naphthodioxin-substituted Pc derivatives. In this paper, α- and β-substituted metal-free Pcs (**4**, **5**) with eight hexyloxy units on the non-peripheral sites along with four benzodioxin (or four naphthodioxin groups) on the peripheral sites were reported. As an extension to this synthetic procedure, the Q-band absorption and aggregation properties of the compounds were also studied. It was observed that substitution of the hexyloxy and phenoxy (or naphthoxy) groups for the protons at the α- and β- positions not only increases the solubility of the Pcs, but also leads to greater bathochromic shifts. Photophysical and fluorescent quenching of compounds **4** and **5** were examined. Additionally, the photophysical properties of the metal-free Pcs were compared with the unsubstituted H_2_Pc. In the literature, there are few studies on the electrochemistry of hexadeca-substituted Pcs [13,26]. Therefore, we decided to study the electrochemical properties of metal-free Pcs (**4**, **5**).

## 2. Results and Discussion

### 2.1. Synthesis and Characterization

**Scheme 1:**
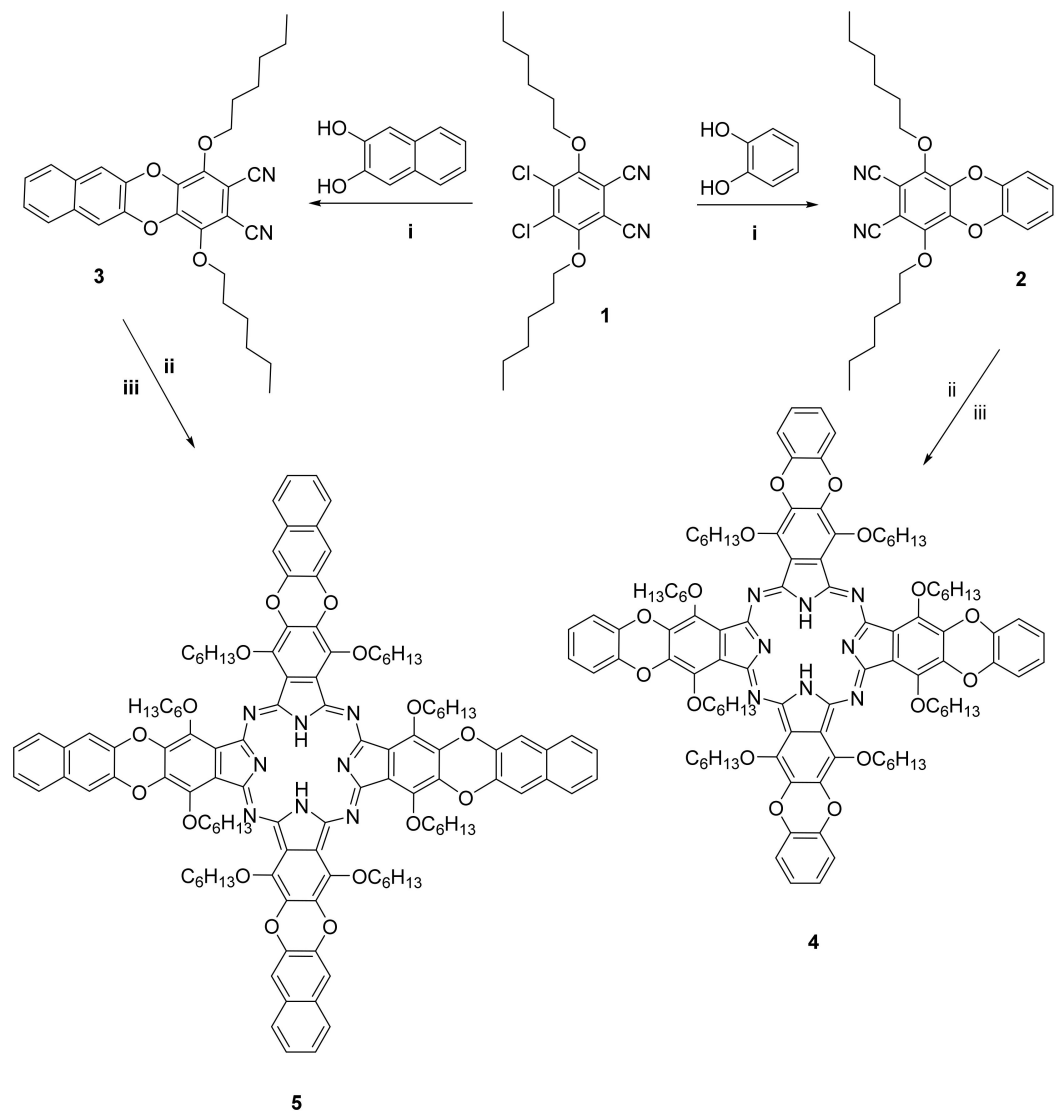
The synthesis of **2**, **3, 4**, and **5**. (**i**) DMF, K_2_CO_3_, room temperature for 24 h and 50 °C for 4 h; (**ii**) hexan-1-ol, metallic Li, 150 °C, 2 h; **(iii**) glacial acetic acid, 0.5 h.

1,4-Bis(hexyloxy)dibenzodioxin-2,3-dicarbonitrile (**2**) and 1,4-bis(hexyloxy)benzo[b]naphtha [2,3-e]dioxin-2,3-dicarbonitrile (**3**) were prepared with aromatic substitution of compound **1** with 1,2-dihydroxybenzene (or 2,3-dihydroxynaphthalene) using potassium carbonate as the base in dry dimethylformamide (Scheme 1).

The most common method for preparing metal-free Pcs is the cyclotetramerization of phthalonitriles in a high boiling solvent (n-pentanol, n-hexanol, or 2-dimethylaminoethanol) in the presence of a base such as 1,8-diazabicycloundec-7-ene (DBU). In the second method, cyclotetramerization is achieved by the reaction of phthalonitrile with a lithium alkoxide, which results in the formation of the corresponding alkaline metal-containing Pc and then it can be reacted with a suitable mineral acid to the metal-free Pc. We employed the second method for the synthesis of **4** and **5**; that is, we cyclized **2** (or **3**) with metallic lithium in 1-hexanol, and acidified with glacial acetic acid to obtain **4** (or **5**).

As expected, the hexadeca-substituted Pcs (**4**, **5**) synthesized during this work were obtained as single isomers. They were purified by column chromatography. Due to the presence of hexyloxy groups, the Pcs **4** and **5** had high solubility in common solvents like dichloromethane (DCM), chloroform, tetrahydrofuran (THF), dimethyl sulfoxide (DMSO), and dimethyl formamide (DMF).

FT-IR, mass, ^13^C NMR, UV–Vis, and ^1^H NMR were employed as the spectroscopic characterization methods of the synthesized compounds. We found out that the spectral results were harmonious with the structures proposed. The stretching vibration of the C≡N group of compound **2** was observed at 2232 cm^−1^, the aliphatic CH stretching was observed in the range of 2952–2869 cm^−1^ as expected, and the CH stretching of the aromatic group was recorded at 3086 cm^−1^, in the IR spectrum. The ^1^H NMR spectrum of **2** in deuterochloroform (CDCl_3_) showed the aromatic protons at δ 7.05 and 6.97 ppm, respectively (Figure S1). The OCH_2_ and OCCH_2_ protons resonated at δ 4.24 and 1.91 ppm as a triplet and a quintet, respectively, and the aliphatic CH_2_ and CH_3_ protons were observed as a quintet at δ 1.54, as a multiplet at δ 1.41, and as a triplet at δ 0.94 ppm. In the ^13^C NMR spectrum of **2** in CDCl_3_, the aromatic and aliphatic carbon atoms appeared in the range of δ 146.6–104.5 ppm and δ 76.0–14.0 ppm, respectively (Figure S2).

In the IR spectrum of **3**, C≡N, aliphatic CH, and aromatic CH stretchings were recorded at 2231, 2955–2860, and 3080 cm^−1^, respectively. ^1^H NMR spectrum of **3** showed the aromatic protons at δ 7.71 (dd), 7.46 (dt), and 7.42 (s) ppm in CDCl_3_ (Figure S3). The protons of hexyloxy groups were determined between δ 4.29 and 0.97 ppm, respectively. In the ^13^C NMR spectrum of **3**, the aromatic and aliphatic carbon atoms appeared in the range of δ 146.7–104.1 ppm and δ 76.1–14.0 ppm, respectively (Figure S4).

An important distinction is the disappearance of sharp nitrile vibrations at 2230 cm^−1^ for all phthalonitriles, and this is an important evidence of cyclotetramerization. Pcs **4** and **5** had similar FT-IR spectra. Aliphatic CH, C-O-C, and inner core NH vibrations were recorded at 2954–2857, 1259–1219, and 3298–3300 cm^−1^, respectively. In the ^1^H NMR spectra of compounds **4** and **5** in CDCl_3_, the aromatic and aliphatic protons resonated between δ 7.21–7.09 ppm and 5.07–0.92 ppm for **4** and between δ 7.82–7.43 ppm and 5.14–0.95 ppm for **5** (Figure S5 and Figure S6). For Pcs **4** and **5**, the inner core protons were monitored at 0.13 ppm and 0.22 ppm, respectively. In the ^1^H NMR spectra, the triplet at 4.24 ppm for OCH_2_ protons in **2** and δ 4.29 ppm in **3** was observed at δ 5.07 ppm (for **4**) and δ 5.14 ppm (for **5**) in Pcs. In the ^13^C NMR spectrum of compound **4**, the protonated aromatic carbon atoms were observed at 124.3 and 116.7 ppm as expected (Figure S7). Unsaturated carbon atoms appeared at 141.7, 140.5, 139.3, and 76.6 ppm, respectively. Aliphatic carbon atoms were observed in the range of 76.6–14.1 ppm. Protonated aromatic carbon atoms of compound **5** were observed at 127.0, 125.6, and 112.7 ppm, respectively (Figure S8). Unsaturated carbon atoms appeared at 141.2, 140.6, 138.7, 131.1, and 76.8 ppm, respectively. Aliphatic carbon atoms of compound **5** were monitored in the range of δ 76.8–14.1 ppm. The molecular ion peaks were observed at *m*/*z* 1738.6 for compound **4** and 1938.6 for **5**, respectively (Figure S9 and Figure S10). The experimental section contains more detailed information for synthesis and characterization.

**Figure 1:**
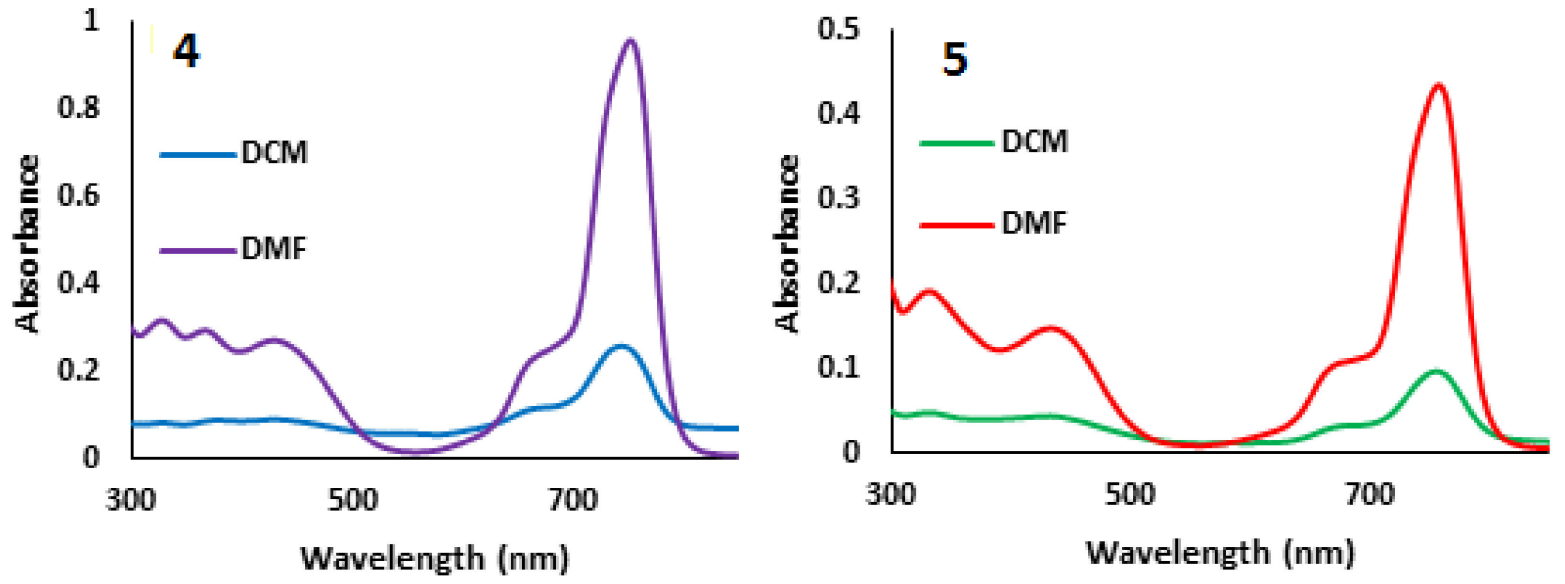
UV-Vis spectra of **4** and **5** in DCM and DMF (concentration: 4 × 10^−6^ M).

In H_2_Pcs and metallo phthalocyanines (MPcs), strong absorption bands are detected between 600–700 nm (visible region), and between 300–350 nm (UV region) [34]. In the UV-Vis spectrum, due to the D_4h_ symmetry, MPc derivatives feature a single Q band, whereas metal-free Pcs show a split Q band owing to the reduction of D_4h_ symmetry to D_2h_. However, the studied hexadeca-substituted metal-free Pcs (**4**, **5**) feature, in the UV-Vis absorption spectra, a single Q band in DMF because of the deprotonated/free basic species in the solvent (Figure 1). Hydrogen bonding of the DMF molecules with the inner NH group may cause deprotonation of the NHs in the ring cavity [35]. The distortion of the macrocycle is probably the reason for the easy deprotonation of Pcs. In DMF, the UV-Vis spectra of **4** and **5** feature a single broad Q band at 743 nm for **4** and 755 nm for **5**. A range of 377–331 nm was observed for the B bands. When compared to the previous studies having naphthoxy (at the peripheral positions)-substituted Pc, hexadeca-substituted H_2_Pc having benzodioxin or naphthodioxin groups at peripheral positions induces a red shift of the Q band about 32 nm for **4** and 44 nm for **5** [26]. For compounds **4** and **5**, UV-Vis absorption spectra were also recorded in both THF and DCM (Figure S11). A broad Q band was detected with higher intensity than that in DMF. However, they did not exhibit an additional absorption band beyond the Q band due to J-aggregation or protonation of meso-nitrogens [36,37,38,39].

**Figure 2:**
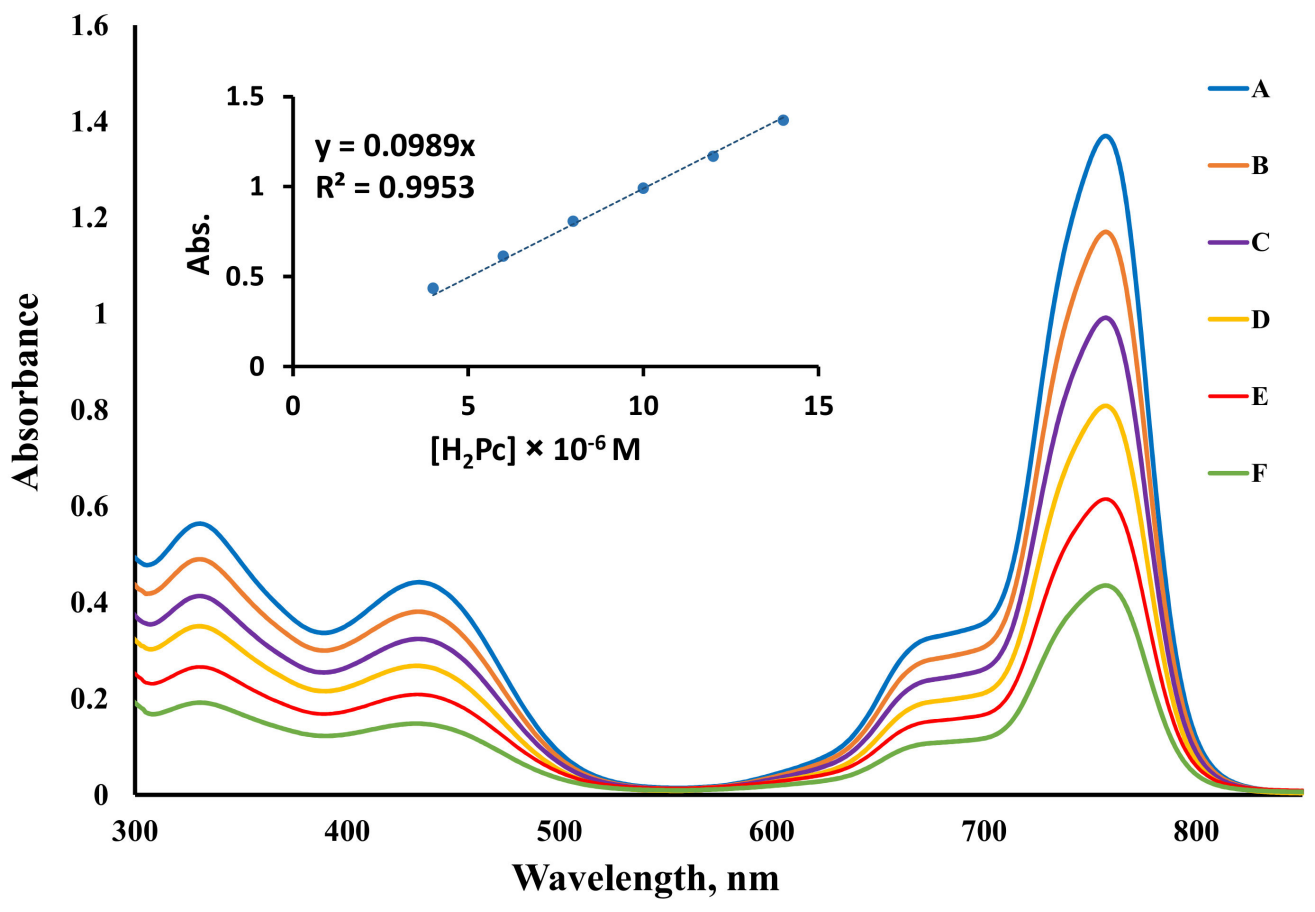
UV-Vis spectra of compound **5** in dichloromethane at different concentrations: (A) 14 × 10^−6^ M, 12 × 10^−6^ M (B), 10 × 10^−6^ M (C), 8 × 10^−6^ M (D), 6 × 10^−6^ M (E), 4 × 10^−6^ M (F).

UV-Vis spectra of the compounds **4** and **5** at different concentrations were recorded in DCM (a series of spectra can be seen in Figure 2 for compound **5**). The Q-band intensity of **4** and **5** increased by increasing concentration and no new bands were observed [40].

### 2.2. Fluorescence Studies

**Figure 3:**
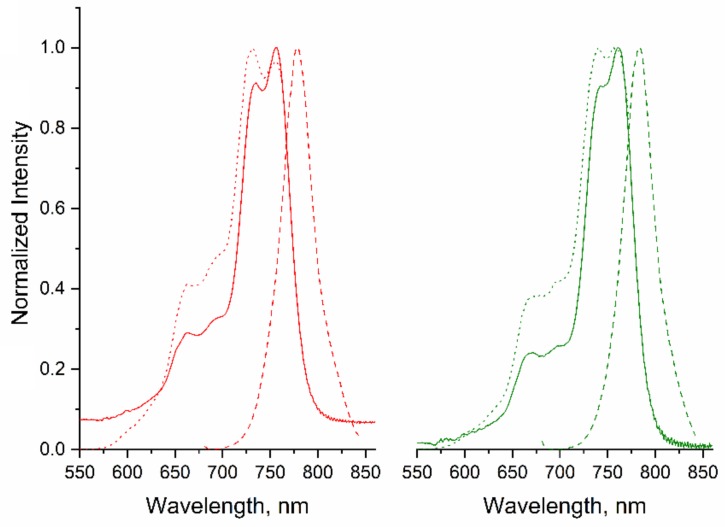
Absorption (solid lines), excitation (dotted lines), and emission spectra (dashed lines) of **4** (red) and **5** (green) in tetrahydrofuran (THF) (4 × 10^−6^ M).

Experiments of fluorescence of **4** and **5** were carried out in THF in accordance with the literature [41,42]. Fluorescent excitation and emission spectra and absorption spectra of **4** and **5** are presented in Figure 3. The fluorescence spectra of **4** and **5** were performed at 671 nm and the emissions were determined at 779 nm for **4** and 783 nm for **5**. The Stokes’ shifts of **4** and **5** were 23 nm and 22 nm, respectively. The Stokes’ shifts were observed to correspond to a usual value of around 20 nm for Pcs [26,41,43]. To calculate the quantum yields (Φ_F_) of **4** and **5**, the comparative method was used and unsubstituted zinc Pc (ZnPc) was standard with Φ_F_ = 0.17 [41,42]. The quantum yield (Φ_F_), natural radiative lifetime (τ_0_), and fluorescence lifetime (τ_F_) values of the synthesized Pc complexes are given in Table 1 in detail. Calculated ΦF values for **4** and **5** were greater than those of unsubstituted ZnPc used as a standard. According to the literature, synthesis and photophysical measurements of α- and β-substituted phthalocyanines were made by Kurt et al. [26]. In another study performed by Awaji et al., octahexyloxy- and octa-(4-trifluoromethoxyphenyl)-substituted Pc synthesis and photophysical measurements were performed [18]. In these studies, the fluorescence quantum yields measured for H_2_Pcs were lower than that of standard ZnPc. As compared to the hexadeca-substituted Pcs in the literature, it was observed that the presence of benzodioxin or naphthodioxin groups increased fluorescence quantum yields of metal-free Pcs.

### 2.3. Benzoquinone (BQ)-Based Fluorescent Quenching

**Figure 4:**
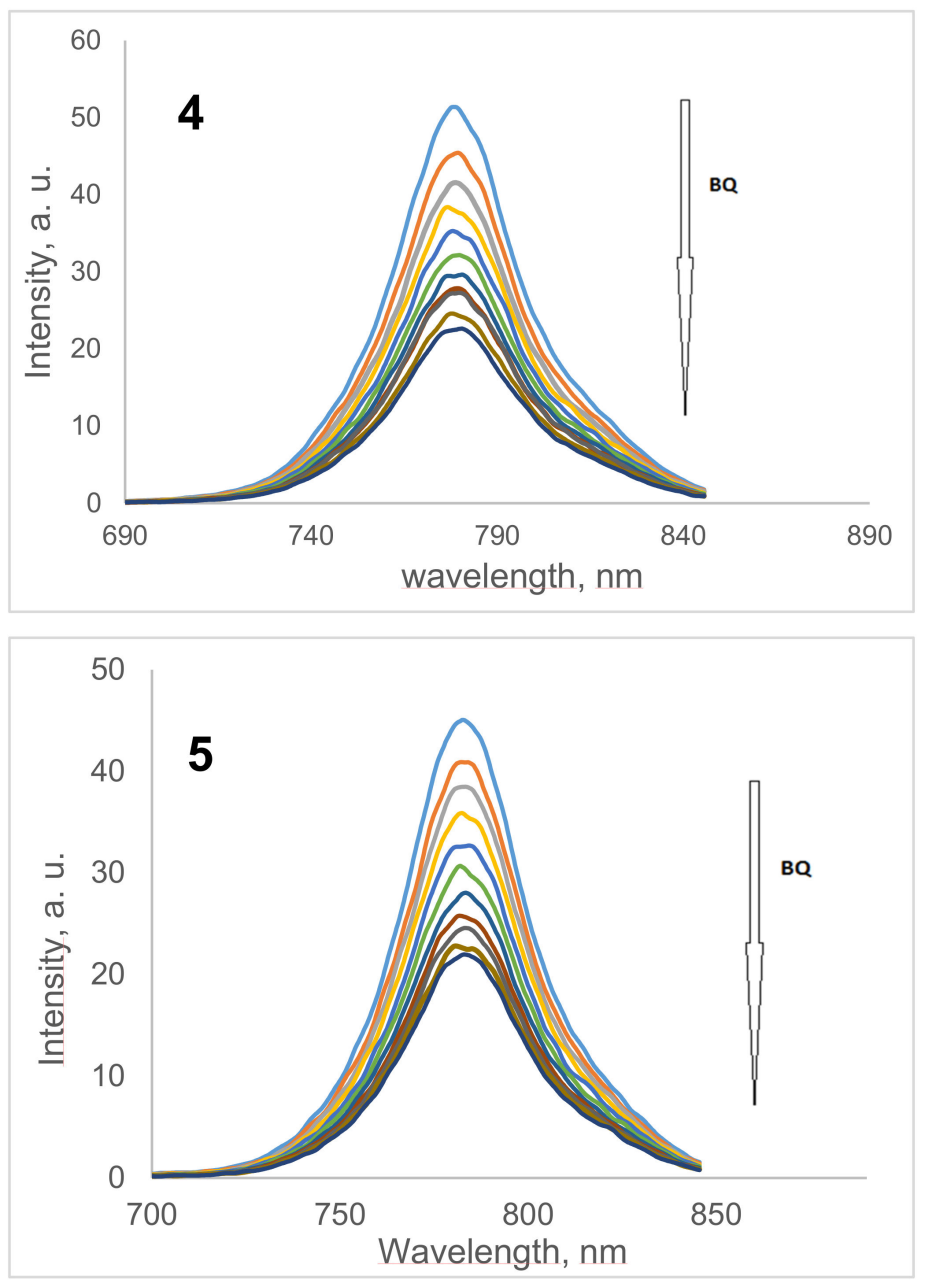
Fluorescence emission spectral changes of **4** (4 × 10^−6^ M) and **5** (4 × 10^−6^ M) in THF upon addition of solutions of BQ in THF at different concentrations. [BQ] = 0.000, 0.004, 0.008, 0.0012, 0.016, …, 0.040 M.

**Figure 5:**
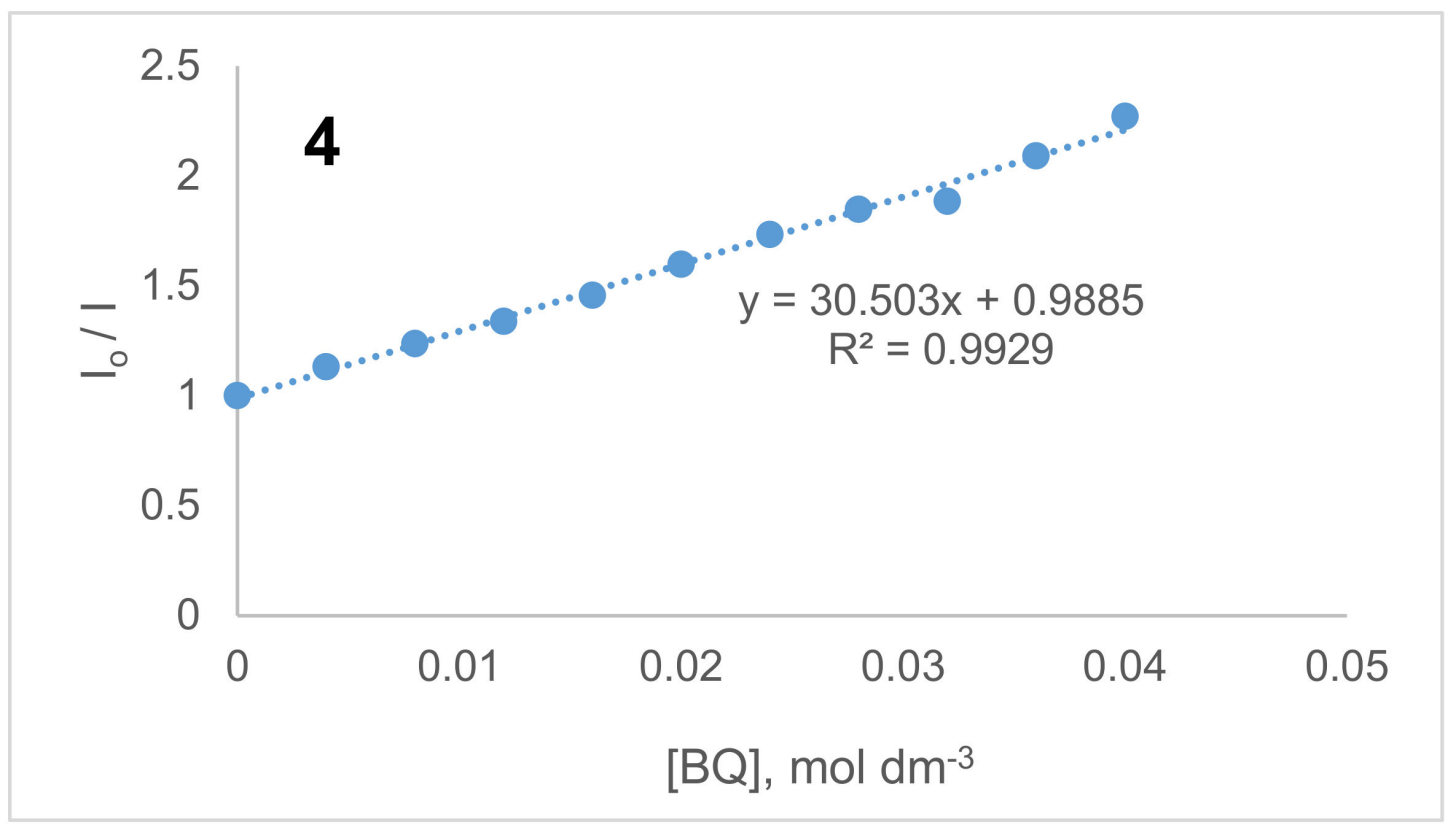

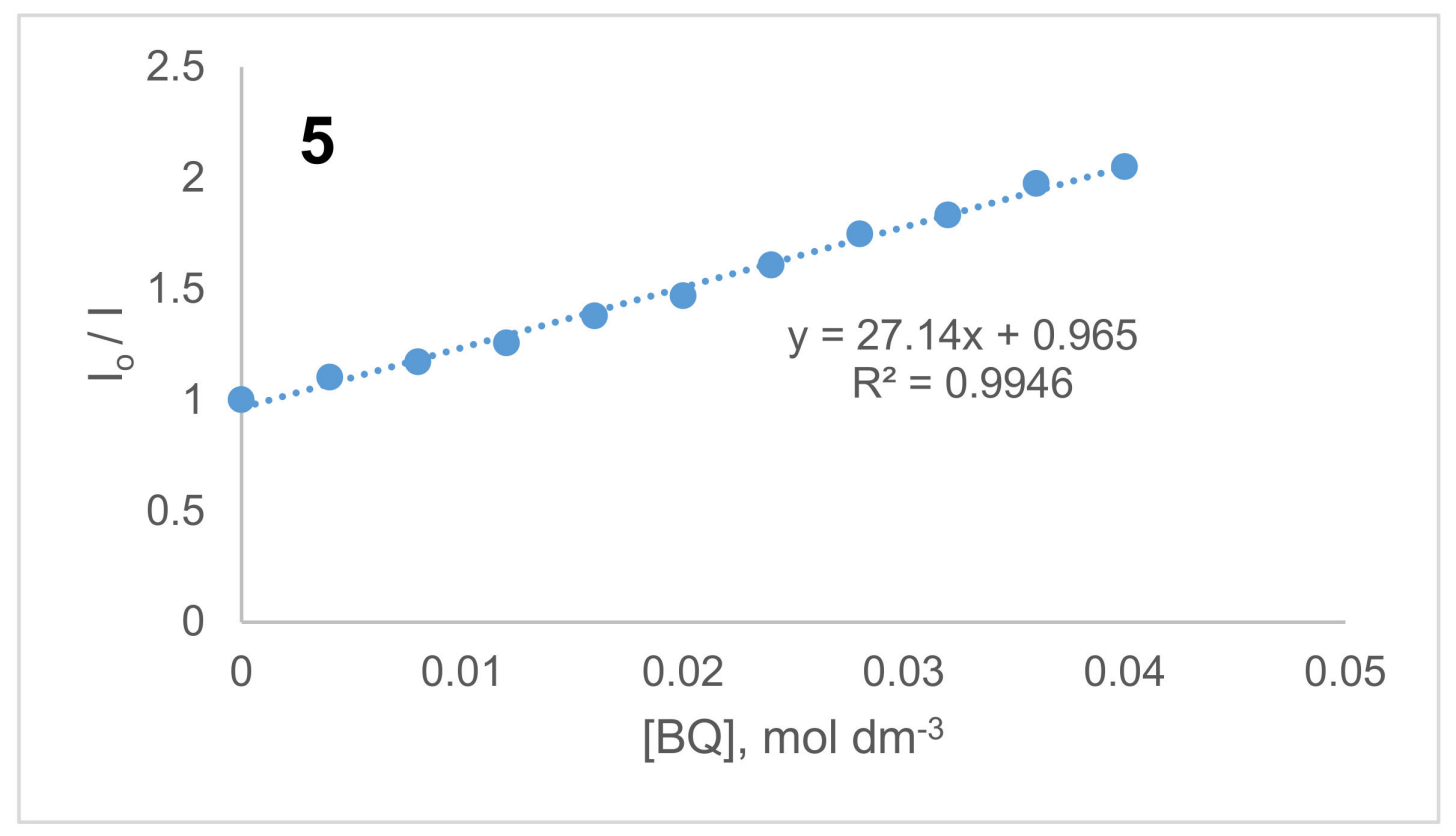
Stern–Volmer plots of 4.00 micromolar solutions of BQ in THF for compounds **4** and **5**.

For compounds **4** and **5**, the fluorescence quenching recorded in THF with BQ was found to be consistent with the Stern–Volmer kinetics. The emission spectra of **4** and **5** with the addition of varying concentrations of BQ are shown in Figure 4. The intensity of **4** (or **5**) decreases in parallel with the increasing concentration of BQ. The slope of the graph studied for Equation (3) is linear. This is consistent with diffusion controlled bimolecular reactions (Figure 5) [41,43]. The obtained K_SV_ and k_q_ (bimolecular quenching constants) values of Pcs are shown in Table 1. The K_sv_ and k_q_ values of **4** and **5** were smaller than those for unsubstituted ZnPc. When BQ was employed as a quencher on excited Pcs as a fluorophore, an energy transfer occurred between the former and the latter. With an increase in BQ concentration, there was a gradual decrease in the fluorescent intensity.

### 2.4. Electrochemical Measurements

**Figure 6:**
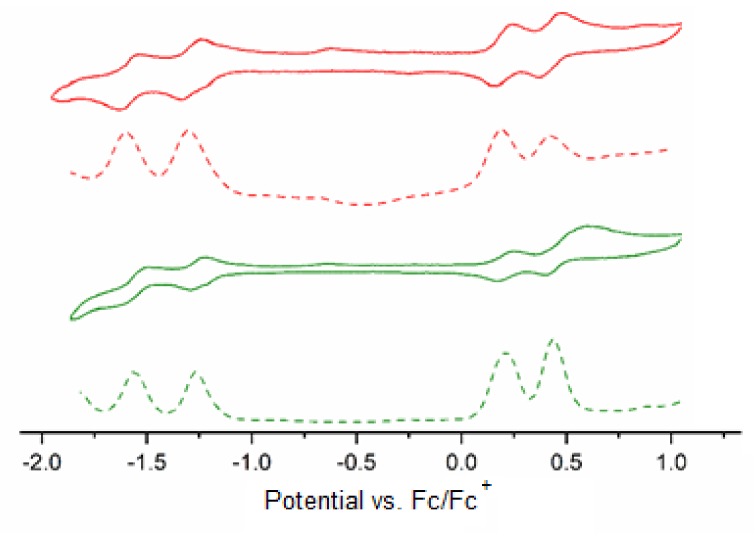
Cyclic (solid lines) and square wave (dashed lines) voltammograms of **4** (red) and **5** (green) (cyclic voltammetry was recorded at the scan rate of *v* = 50 mV·s^−1^).

The electrochemical behavior of **4** and **5** was investigated with cyclic voltammetry (CV) and square wave voltammetry (SWV) in electrochemical grade dichloromethane containing 0.1 M tetrabutylammonium perchlorate as the electrolyte (Figure 6), and the half-wave potentials are presented in Table 2. As can be seen, due to the Pc-based redox processes, **4** and **5** exhibited two reversible oxidations and two reversible reductions [44,45]. The first oxidation peak of **5** was 60 mV positively shifted in comparison with **4** as the result of the effect of the naphthalene ring. The HOMO level of **4** and **5** was calculated from the first oxidation, indicating the effective destabilization of the HOMO energy level which is caused by the introduction of different groups at the non-peripheral and peripheral positions of the Pc [20,26,28]. The optical band gap was calculated to be 1.58 and 1.56 eV for **4** and **5**, respectively, which is slightly higher than the electrochemical band gap. In addition, the HOMO and LUMO energy values suggest that the compounds can be viewed as candidates for organic semiconductors [46,47].

## 3. Materials and Methods

### 3.1. Materials and Equipment

Commercial suppliers were contacted for acquiring all solvents and reagents used in this study. Solvents were stored using molecular sieves. An Agilent VNMRS 500 MHz spectrometer (Agilent, Santa Clara, CA, USA) was used to obtain ^1^H and ^13^C NMR spectra of all new compounds. A PerkinElmer Spectrum One FT-IR spectrometer (PerkinElmer, Waltham, MA, USA) was used to obtain the FT-IR spectra of the compounds and a Scinco LabProPlus UV/Vis spectrophotometer (Scinco, Seoul, Korea) was used to record the UV-Vis spectra. The UV/Vis spectrophotometer has a spectral range of 300–800 nm and the path length of the conventional quartz cuvette used is 1 cm. A PerkinElmer LS55 fluorescence spectrophotometer (PerkinElmer, Waltham, MA, USA) was used to study fluorescent properties. A Bruker Microflex matrix-assisted laser desorption-ionization (MALDI) time-of-flight (TOF) mass spectrometer (Bruker Daltonics, Bremen, Germany) was employed to acquire the mass spectra of compounds. An agreement was found between the isotopic patterns for all assigned signals and the calculated natural abundance. 4,5-Dichloro-3,6-bis(hexyloxy)phthalonitrile (**1**) as the starting compound, was synthesized following a previously reported procedure [27].

The square wave voltammetry (SWV) and cyclic voltammetry (CV) experiments were carried out with a Gamry Reference 600 potentiostat/galvanostat (Warminster, PA, USA) using a working electrode of glassy carbon (surface area: 0.071 cm^2^), a counter electrode of platinum wire, and a reference electrode of saturated calomel (SCE). Ferrocene was used as an internal standard. Tetrabutylammonium perchlorate (TBAP) in DCM) was used as the supporting electrolyte at a concentration of 0.10 mol dm^−3^.

### 3.2. Chemistry

#### 3.2.1. 1,4-Bis(hexyloxy)dibenzodioxin-2,3-dicarbonitrile (**2**)

A 2.00 g portion of compound **1** (5.03 mmol) was dissolved under nitrogen atmosphere using 50 mL of dry DMF. After all phthalonitrile was dissolved, 0.55 g of catechol (1,2-dihydroxybenzene) (5.03 mmol) and 2.09 g of K_2_CO_3_ (15.10 mmol) were added to the solution altogether. The reaction mixture was stirred at room temperature for 24 h and then at 50 °C for a further 4 h. The mixture was then poured into 150 mL of crushed ice water mixture. The crude product was extracted with chloroform, dried with Na_2_SO_4_. Purification was carried out by crystallization from ethanol. C_26_H_30_N_2_O_4_ yield: 1.44 g (66.0%); mp: 74 °C. FT-IR (υ_max_, cm^−1^): 3086 (Ar–H), 2952–2869 (Aliph–CH), 2232 (C≡N), 1110 (C–O–C). ^1^H NMR (CDCl_3_): δ, ppm: 7.03 (dd, *J* = 7.4, 3.7 Hz, 2H, Ar–H), 6.95 (dd, *J* = 6.0, 3.6 Hz, 2H, Ar–H), 4.22 (t, *J* = 6.5 Hz, 4H, O–CH_2_), 1.84 (m, 4H, CH_2_), 1.53 (dt, *J* = 14.68, 7.2 Hz, 4H, CH_2_), 1.37 (m, 8H, CH_2_), 0.93 (t, *J* = 6.86 Hz,6H, CH_3_). ^13^C NMR (CDCl_3_): δ, ppm: 146.6, 140.8, 140.0, 125.6, 116.8, 112.6, 104.5, 76.0, 31.4, 29.9, 25.3, 22.5, 14.0. MS: (EI) *m*/*z* 434.19 [M]^+^.

#### 3.2.2. 1,4-Bis(hexyloxy)benzo[b]naphtho[2–e]dioxin-2,3-dicarbonitrile (**3**)

A 2.00 g portion of compound **1** (5.03 mmol) and 0.806 g of 2,3-dihydroxynaphthalene (5.03 mmol) were dissolved under nitrogen atmosphere using 50 mL of dry DMF. A 2.09 g portion of anhydrous K_2_CO_3_ (15.10 mmol) was added and the reaction mixture was stirred vigorously at room temperature for 24 h, then the reaction temperature was raised to 50 °C. Reaction continued at this temperature for a further 4 h. The mixture of reaction was poured into approximately 150 mL of iced water, extracted with chloroform, and the organic phase was dried with Na_2_SO_4_. Purification of the crude product was carried out by crystallization with ethanol. C_30_H_32_N_2_O_4_ yield: 1.38 g (56.8%); mp: 152 °C. FT-IR (υ_max_, cm^−1^): 3080 (Ar–H), 2955–2860 (Aliph–CH), 2231 (C≡N), 1080 (C–O–C). ^1^H NMR (CDCl_3_): δ, ppm: 7.70 (dd, *J* = 6.2, 3.3 Hz, 2H, Ar–H), 7.44 (dt, *J* = 6.2, 3.4 Hz, 2H, Ar–H), 7.38 (s, 2H, Ar–H), 4.28 (t, *J* = 6.5 Hz, 4H, O–CH_2_), 1.88 (dt, *J* = 14.9, 6.7 Hz, 4H, CH_2_), 1.58 (quintet, *J* = 7.4 Hz, 4H, CH_2_), 1.40 (m, 8H, CH_2_), 0.95 (m, 6H, CH_3_). ^13^C NMR (CDCl_3_): δ, ppm: 146.7, 140.1, 139.0, 131.1, 127.1, 126.5, 113.3, 112.6, 104.1, 76.1, 31.4, 29.9, 25.3, 22.5, 14.0.MS: (EI) *m*/*z* 484.05 [M]^+^.

#### 3.2.3. 1,4,8,11,15,18,22,25-Octakis(Hexyloxy)-tetra(benzodioxin) Phthalocyanine (**4**)

A 0.09 g portion of metallic lithium (13.8 mmol) was dissolved under nitrogen atmosphere in 5 mL of n-hexanol at 80 °C. To this solution was added 0.3 g of 1,4-bis(hexyloxy)dibenzo[b,e] [1,4]dioxin-2,3-dicarbonitrile (2) (0.69 mmol) and heated at 150 °C for 2 h. After cooling the reaction mixture to room temperature, it was poured into 50 mL of ethanol, 3 mL of glacial acetic acid was added, stirred, and the precipitate formed was filtered to give the crude product. With the addition of the acid, the resulting Li**_2_**Pc was converted to H**_2_**Pc. After repeated washing with methanol, the precipitate was purified by column chromatography over silica gel (eluent: CH_2_Cl_2_). C_104_H_122_N_8_O_16_ yield: 0.15 g (57%); mp: >200 °C; FT-IR (υ_max_, cm^−1^): 3298 (Ar–H), 2954–2857 (Aliph–CH), 1082 (C–O–C). ^1^H NMR (CDCl_3_): δ, ppm: 7.20 (dd, *J* = 5.9, 3.7 Hz, 8H, Ar–H), 7.09 (dd, *J* = 6.0, 3.5 Hz, 8H, Ar–H), 5.06 (t, *J* = 6.6 Hz,16H, O–CH_2_), 2.10 (quintet, *J* = 7.0 Hz, 16H, CH_2_), 1.72 (quintet, *J* = 7.4 Hz, 16H, CH_2_), 1.40 (m, 32H, CH_2_), 0.91 (t, *J* = 6.8 Hz, 24H, CH_3_), 0.13 (bs, 2H, NH). ^13^C NMR (CDCl_3_): δ, ppm: 141.7, 140.5, 139.3, 124.3, 116.7, 76.6, 31.9, 30.3, 26.0, 22.8, 14.1. UV-Vis (DMF): λ_max_, nm (log ε): 377 (4.34), 743 (4.81). UV-Vis (DMF): λ_max_, nm (log ε): 377 (4.34), 743 (4.81). MS: (MALDI-TOF) *m*/*z* 1738.6 [M]^+^. Anal. calc. for C_104_H_122_N_8_O_16_: C, 71.78%; H, 7.07%; N, 6.44%. Found: C, 71.69%; H, 7.00%; N, 6.35%.

A 0.09 g portion of metallic lithium (13.8 mmol) was dissolved under nitrogen atmosphere in 5 mL of n-hexanol at 80 °C. To this solution was added 0.3 g of 1,4-bis(hexyloxy)dibenzo[b,e] [1,4]dioxin-2,3-dicarbonitrile (2) (0.69 mmol) and heated at 150 °C for 2 h. After cooling the reaction mixture to room temperature, it was poured into 50 mL of ethanol, 3 mL of glacial acetic acid was added, stirred, and the precipitate formed was filtered to give the crude product. With the addition of the acid, the resulting Li**_2_**Pc was converted to H**_2_**Pc. After repeated washing with methanol, the precipitate was purified by column chromatography over silica gel (eluent: CH_2_Cl_2_). C_104_H_122_N_8_O_16_ yield: 0.15 g (57%); mp: >200 °C; FT-IR (υ_max_, cm^−1^): 3298 (Ar–H), 2954–2857 (Aliph–CH), 1082 (C–O–C). ^1^H NMR (CDCl_3_): δ, ppm: 7.20 (dd, *J* = 5.9, 3.7 Hz, 8H, Ar–H), 7.09 (dd, *J* = 6.0, 3.5 Hz, 8H, Ar–H), 5.06 (t, *J* = 6.6 Hz,16H, O–CH_2_), 2.10 (quintet, *J* = 7.0 Hz, 16H, CH_2_), 1.72 (quintet, *J* = 7.4 Hz, 16H, CH_2_), 1.40 (m, 32H, CH_2_), 0.91 (t, *J* = 6.8 Hz, 24H, CH_3_), 0.13 (bs, 2H, NH). ^13^C NMR (CDCl_3_): δ, ppm: 141.7, 140.5, 139.3, 124.3, 116.7, 76.6, 31.9, 30.3, 26.0, 22.8, 14.1. UV-Vis (DMF): λ_max_, nm (log ε): 377 (4.34), 743 (4.81). UV-Vis (DMF): λ_max_, nm (log ε): 377 (4.34), 743 (4.81). MS: (MALDI-TOF) *m*/*z* 1738.6 [M]^+^. Anal. calc. for C_104_H_122_N_8_O_16_: C, 71.78%; H, 7.07%; N, 6.44%. Found: C, 71.69%; H, 7.00%; N, 6.35%.

A 0.09 g portion of metallic lithium (13.8 mmol) was dissolved under nitrogen atmosphere in 5 mL of n-hexanol at 80 °C. To this solution was added 0.3 g of 1,4-bis(hexyloxy)dibenzo[b,e] [1,4]dioxin-2,3-dicarbonitrile (2) (0.69 mmol) and heated at 150 °C for 2 h. After cooling the reaction mixture to room temperature, it was poured into 50 mL of ethanol, 3 mL of glacial acetic acid was added, stirred, and the precipitate formed was filtered to give the crude product. With the addition of the acid, the resulting Li**_2_**Pc was converted to H**_2_**Pc. After repeated washing with methanol, the precipitate was purified by column chromatography over silica gel (eluent: CH_2_Cl_2_). C_104_H_122_N_8_O_16_ yield: 0.15 g (57%); mp: >200 °C; FT-IR (υ_max_, cm^−1^): 3298 (Ar–H), 2954–2857 (Aliph–CH), 1082 (C–O–C). ^1^H NMR (CDCl_3_): δ, ppm: 7.20 (dd, *J* = 5.9, 3.7 Hz, 8H, Ar–H), 7.09 (dd, *J* = 6.0, 3.5 Hz, 8H, Ar–H), 5.06 (t, *J* = 6.6 Hz,16H, O–CH_2_), 2.10 (quintet, *J* = 7.0 Hz, 16H, CH_2_), 1.72 (quintet, *J* = 7.4 Hz, 16H, CH_2_), 1.40 (m, 32H, CH_2_), 0.91 (t, *J* = 6.8 Hz, 24H, CH_3_), 0.13 (bs, 2H, NH). ^13^C NMR (CDCl_3_): δ, ppm: 141.7, 140.5, 139.3, 124.3, 116.7, 76.6, 31.9, 30.3, 26.0, 22.8, 14.1. UV-Vis (DMF): λ_max_, nm (log ε): 377 (4.34), 743 (4.81). UV-Vis (DMF): λ_max_, nm (log ε): 377 (4.34), 743 (4.81). MS: (MALDI-TOF) *m*/*z* 1738.6 [M]^+^. Anal. calc. for C_104_H_122_N_8_O_16_: C, 71.78%; H, 7.07%; N, 6.44%. Found: C, 71.69%; H, 7.00%; N, 6.35%.

#### 3.2.4. 1,4,8,11,15,18,22,25-Octakis(Hexyloxy)-tetra(naphthodioxin) Phthalocyanine (**5**)

A 0.09 g portion of metallic lithium (12.2 mmol) was heated in 5 mL of n-hexanol and cooled to room temperature. To this solution was added 0.3 g of 1,4-bis(hexyloxy)benzo[b]naphtho[2,3-e] [1,4]dioxin-2,3-dicarbonitrile (3) (0.62 mmol) and the reaction mixture was stirred and heated at 150 °C. The reaction was continued at this temperature for 2 h. After the mixture was cooled to room temperature, 50 mL of methanol and 3 mL of glacial acetic acid were added, respectively. The resulting green precipitate was filtered off, washed with a considerable amount of methanol. Purification of the crude product was accomplished by column chromatography over silica gel (eluent: CH_2_Cl_2_). Yield: 0.14 g (49%); mp: >200 °C; FT-IR (υ_max_, cm^−1^): 3300 (Ar–H), 2923–2862 (Aliph–CH), 1080 (C–O–C). ^1^H NMR (CDCl_3_): δ, ppm: 7.81 (dd, *J* = 6.1, 3.3 Hz, 8H, Ar–H), 7.63 (m, 8H, Ar–H), 7.46 (m, 8H, Ar–H), 5.13 (t, *J* = 6.6 Hz, 16H, O–CH_2_), 2.16 (quintet, *J* = 6.9 Hz, 16H, CH_2_), 1.79 (quintet, *J* = 7.5 Hz, 16H, CH_2_), 1.45 (m, 32H, CH_2_), 0.94 (t, *J* = 6.8 Hz, 24H, CH_3_), 0.22 (bs, 2H, NH). ^13^C NMR (CDCl_3_): δ, ppm: 141.2, 140.6, 138.7, 131.1, 127.0, 125.6, 112.7, 76.8, 32.0, 30.4, 26.0, 22.8, 14.1. UV-Vis (DMF): λ_max_, nm (log ε): 331 (4.08), 755 (5.38). MS: (MALDI-TOF) *m*/*z* 1938.6 [M]^+^. Anal. calc. for C_120_H_130_N_8_O_16_: C, 74.28%; H, 6.75%; N, 5.77%. Found: C, 74.12%; H, 6.69%; N, 5.71%.

### 3.3. Photophysics

#### 3.3.1. Fluorescent Quantum Yields and Lifetimes

The synthesized Pcs’ (**4**, **5**) fluorescent quantum yields (Φ_F_) were calculated with the comparative method using Equation (1) [27,33,35]:

(1) $$\emptyset_{F}=\emptyset_{F}\left(\mathrm{Std}\right)\frac{F\;\times {\;A}_{\mathrm{Std}}\;\times {\;n}^{2}}{F_{\mathrm{Std}\;\times }A\;\times n_{\mathrm{Std}}^{2}}$$

where F_Std_ and F represent the areas under the fluorescence emission curves of the standard and the samples, respectively; A_Std_ represents the absorbance values of the standard and A represents the synthesized Pcs; and η_Std_ (*ηDMF* = 1.430) and η (*ηTHF* = 1.407) are the refractive indices of the solvents for the standard and the sample, respectively. The unsubstituted ZnPc was used as the reference compound, and the Φ_F_ value in DMF of standard ZnPc is 0.17 [33,34].

PhotochemCAD 2.1 software package was used to calculate the coefficient of natural radiative lifetimes (τ_0_). The fluorescence lifetimes (τF) were estimated using Equation (2)

(2) $$\emptyset_{F}=\frac{\tau_{F}}{\tau_{0}}$$

#### 3.3.2. Fluorescent Quenching Based on Benzoquinone (BQ)

Fluorescent quenching assays for newly synthesized Pcs (**4**, **5**) were performed in THF with the addition of varying concentrations of BQ (as a quencher) ranging from 0.008 to 0.040 M. The fluorescent results measured after the addition of BQ were recorded to determine the constant K_sv_ (Stern–Volmer constant). K_sv_ values were calculated by the ratios of I_o_/I plotted against the concentration of BQ using Equation (3) below, where K_sv_ was calculated from the slope of the line:

(3) $$\frac{I_{0}}{I}=1+K_{\mathrm{SV}}\left[\mathrm{BQ}\right]$$

Bimolecular quenching constants (k_q_) were determined with the newly obtained K_SV_ value using Equation (4):

*K_SV_* = k_q_ × τ_F_ (4)

## 4. Conclusions

In the present study, two novel hexadeca-substituted metal-free Pcs (**4**, **5**) bearing, at non-peripheral positions, eight hexyloxy groups and four benzodioxin (or four naphthodioxin) groups at peripheral positions were prepared. Characterization was performed by different spectroscopic methods. Synthesized compounds showed full compliance with the proposed structures. Using unsubstituted ZnPc as a standard compound, the photophysical properties of **4** and **5** were investigated and compared. The fluorescent quantum yields and lifetimes of compounds **4** and **5** were higher than the standard. In comparison with the other hexadeca-substituted Pcs in the literature, it was found that the presence of benzodioxin or naphthodioxin groups increased fluorescent quantum yields of metal-free Pcs. Furthermore, the fluorescence quenching studies were performed by benzoquinone, a conventional quencher. Additionally, in THF, the Stern–Volmer kinetics were also investigated. When compared with the standard, compounds **4** and **5** showed lower K_sv_ and k_q_ values. For compounds **4** and **5**, the photophysical and electrochemical properties indicate the potential application from organic semiconductors to medicinal agents.

## Figures and Tables

**Table 1:** Excitation, emission spectral data, photophysical parameters, and fluorescent quenching of the **4**, **5** and unsubstituted zinc phthalocyanine (ZnPc).

Compound	Excitation λ_Ex_ (nm)	Emission λ_Em_ (nm)	Stokes’ Shift ΔStokes (nm)	Φ_F_	τ_F_ (ns)	τo (ns)	k_F_ (s^1^) (×10^8^) ^a^	K_sv_^c^	k_q_ (×10^10^ s^−1^) ^c^
**4**	731,755	779	23	0.29	2.09	7.12	1.40	30.50	1.46
**5**	740,756	783	22	0.44	4.25	9.59	1.04	27.14	0.64
**ZnPc** ^b^	670	676	6	0.17	1.03	6.05	16.53	57.60	5.59

^a^ k_F_, fluorescent rate constant. The values were obtained by plugging them into the formula *k_F_* = Φ_F_/τ_F_. ^b^ Adapted from [34]. ^c^ k_q_, bimolecular quenching constants; K_sv_, Stern–Volmer constant.

**Table 2:** Electrochemical properties of the H_2_Pcs (**4**, **5**).

Compound	E_2_^red^ (V)	E_1_^red^ (V)	E_1_^Ox^ (V)	E_2_^Ox^ (V)	HOMO ^a^ (eV)	LUMO ^c^ (eV)	Eg ^b^ (eV)	Eg ^d^ (eV)
**4**	−1.58	−1.3	0.20	0.44	−5.0	−3.42	1.58	1.50
**5**	−1.54	−1.25	0.26	0.48	−5.06	−3.50	1.56	1.51

^a^ Highest occupied molecular orbital (referred to as the HOMO) was calculated by considering the first oxidation potentials. HOMO = − 4.8 − E_1/2_ (vs. Fc/Fc^+^), where 4.8 eV is the energy level of Fc/Fc^+^ below the vacuum level. ^b^ From the onset of absorption spectra, Eg was calculated from the onset of **4** = 786, **5** = 793. Eg was calculated from the absorption onset wavelength (λonset) using Eg = 1240/λonset. ^c^ Lowest unoccupied molecular orbital (referred to as the LUMO) is the sum of HOMO and Eg. ^d^ Eg was calculated from the CV results using first and second oxidation.

## Supplementary Materials

The supplementary materials are available online: Figure S1: ^1^H-NMR spectrum of compound **2** in CDCl_3_, Figure S2: ^13^C-NMR spectrum of compound **2** in CDCl_3_, Figure S3: ^1^H-NMR spectrum of compound **3** in CDCl_3_, Figure S4: ^13^C-NMR spectrum of compound **3** in CDCl_3_, Figure S5: ^1^H-NMR spectrum of compound **4** in CDCl_3_, Figure S6: ^1^H-NMR spectrum of compound **5** in CDCl_3_, Figure S7:^13^C-NMR spectrum of compound **4** in CDCl_3_, Figure S8: ^13^C-NMR spectrum of compound **5** in CDCl_3_, Figure S9: MALDI-TOF mass spectrum of compound **4**, Figure S10: MALDI-TOF mass spectrum of compound **5**, Figure S11: UV-Vis spectra of **4** and **5** in THF (concentration: 4 × 10^−6^ M).
