# Supplementary material for: α- and β-Substituted Metal-Free Phthalocyanines: Synthesis, Photophysical and Electrochemical Properties
Source: Molecules. 2020 Jan 16;25(2):363. doi: 10.3390/molecules25020363 (PMC7024180; doi:10.3390/molecules25020363)
Supplement: Supplementary file 1 [file molecules-25-00363-s001.pdf]

Supplementary Information

α- and β-Substituted Metal Free Phthalocyanines: Synthesis, Photophysical and Electrochemical Properties

Hande P. Karaoğlu[1]* and Ayfer Kalkan Burat[1]*

[1] Istanbul Technical University, Department of Chemistry, TR34469, İstanbul, Turkey; pekbelgin@itu.edu.tr (H.P.K.); kalkanayf@itu.edu.tr (A.K.B.)

* Correspondence: pekbelgin@itu.edu.tr; kalkanayf@itu.edu.tr; Tel.: +90-212-285-32-36

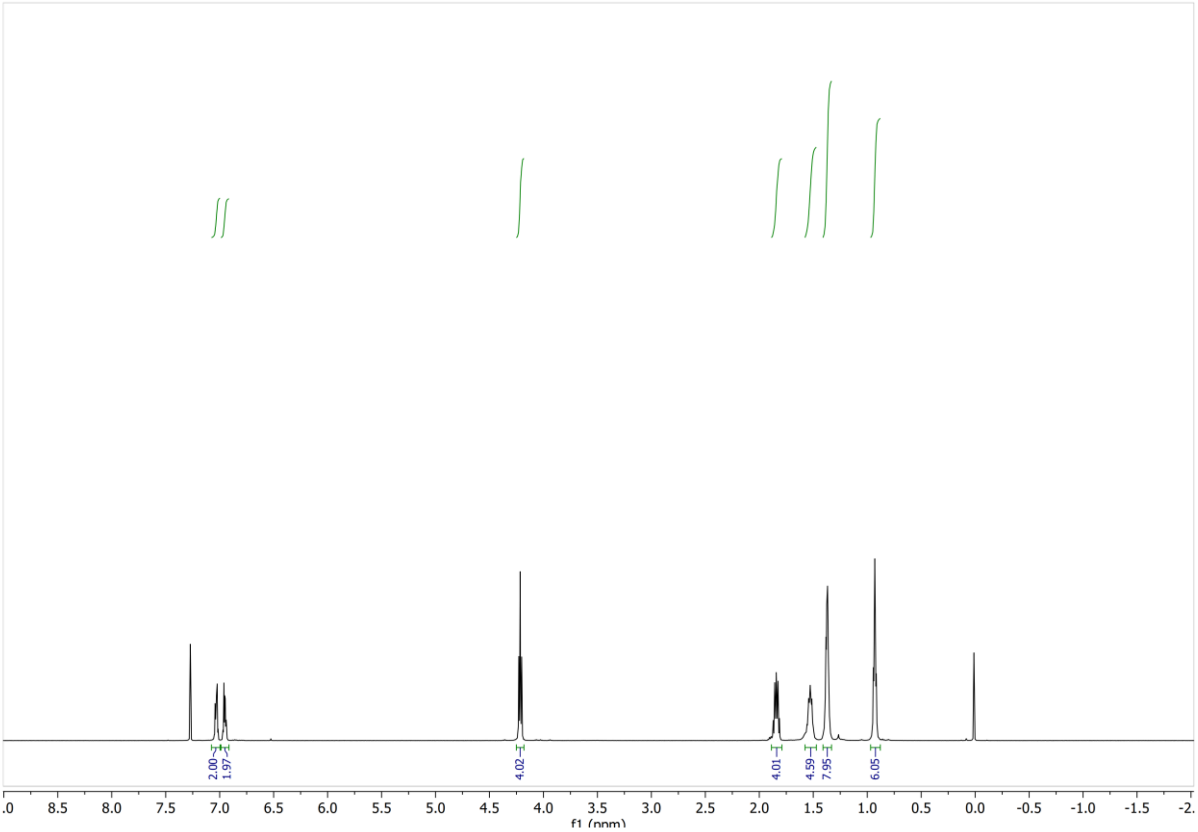

Figure S1. ^{1}H NMR spectrum of **2** in $CDCl_3$.

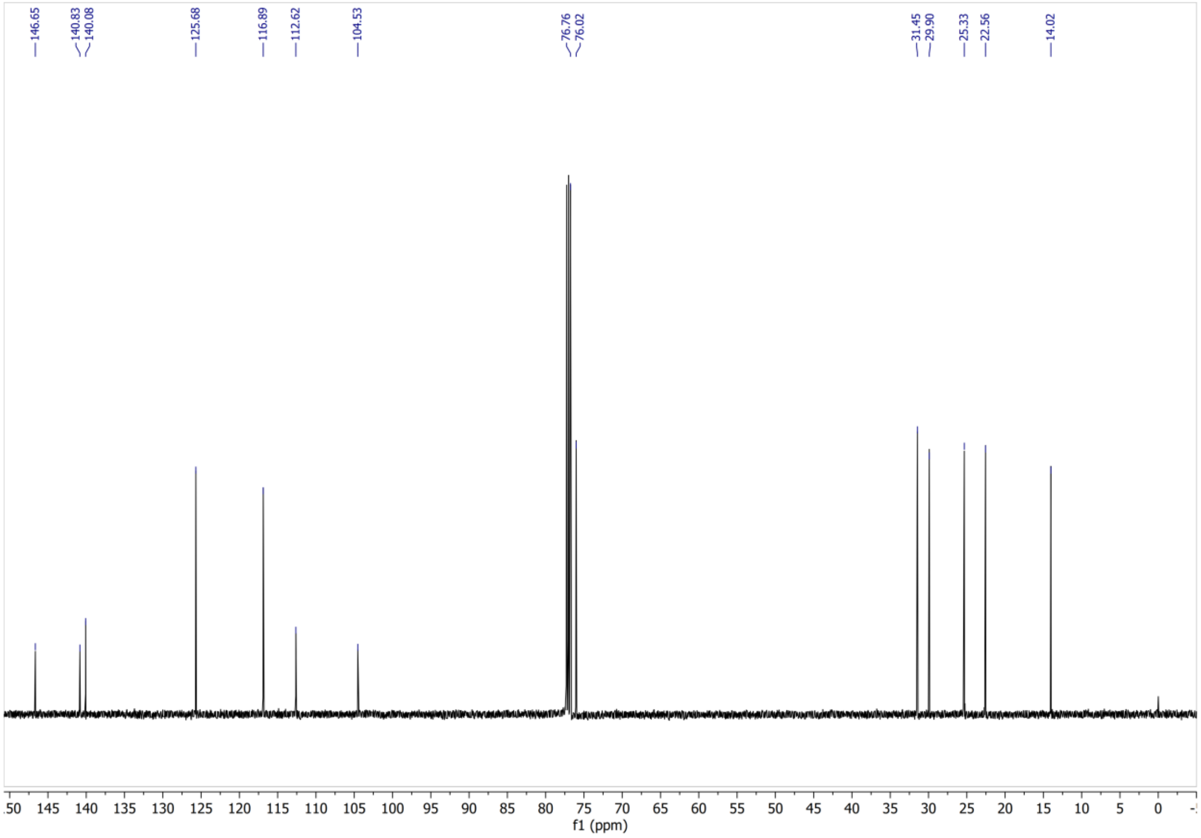

Figure S2. ^{13}C NMR spectrum of **2** in $CDCl_3$.

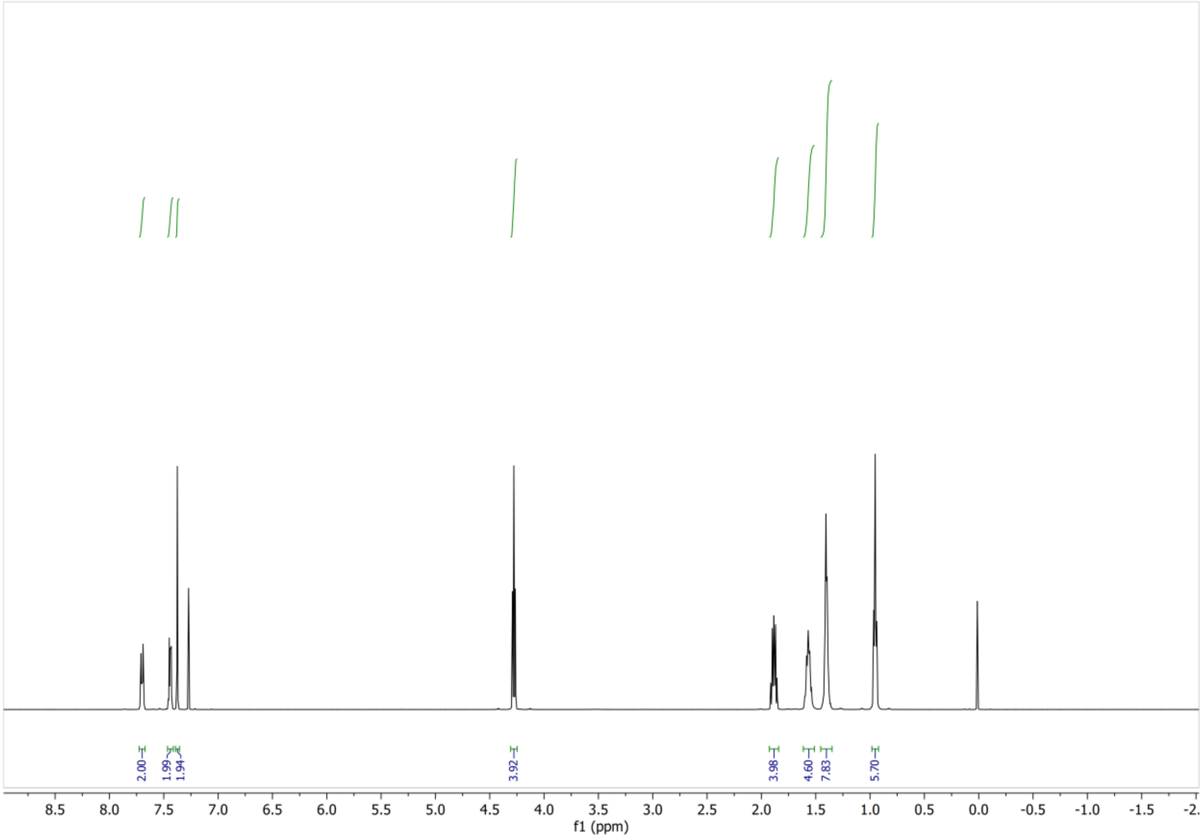

Figure 3. ^{1}H NMR spectrum of **3** in $CDCl_3$.

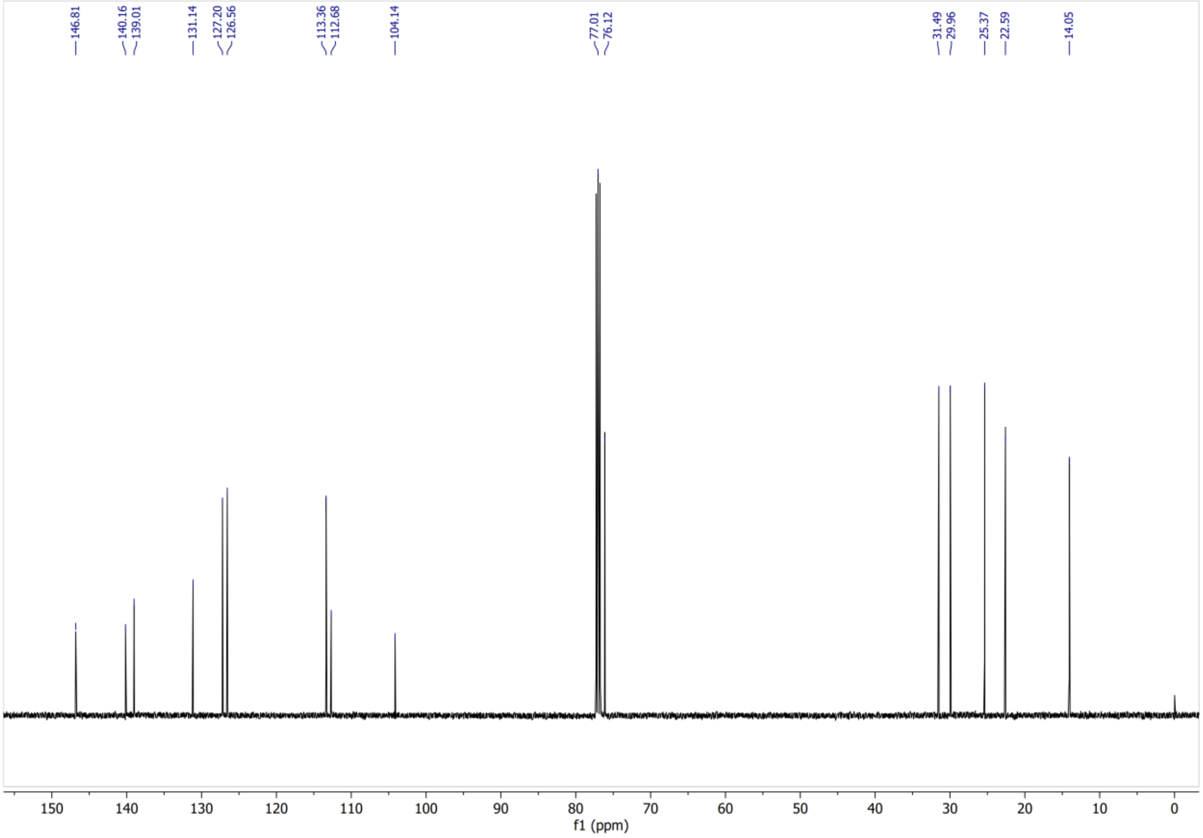

Figure S4. ^{13}C NMR spectrum of **3** in $CDCl_3$.

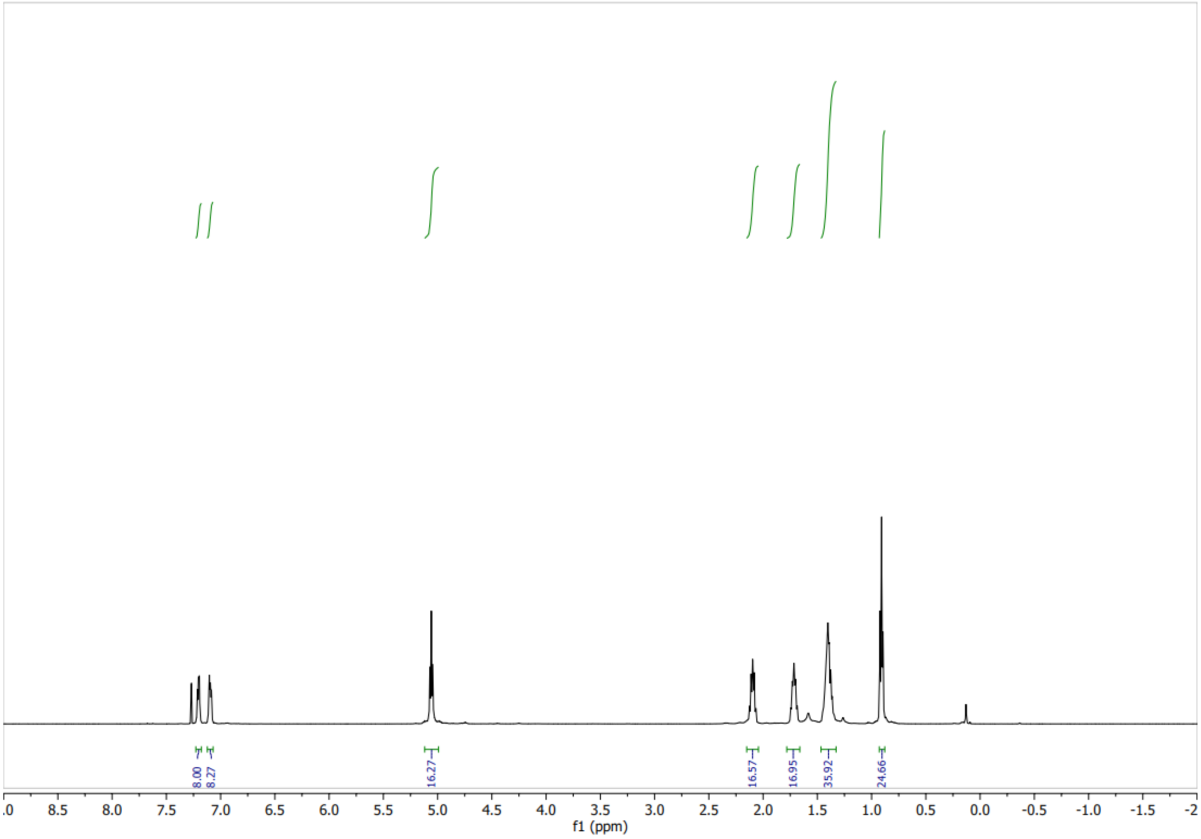

Figure S5. ^{1}H NMR spectrum of **4** in $CDCl_3$.

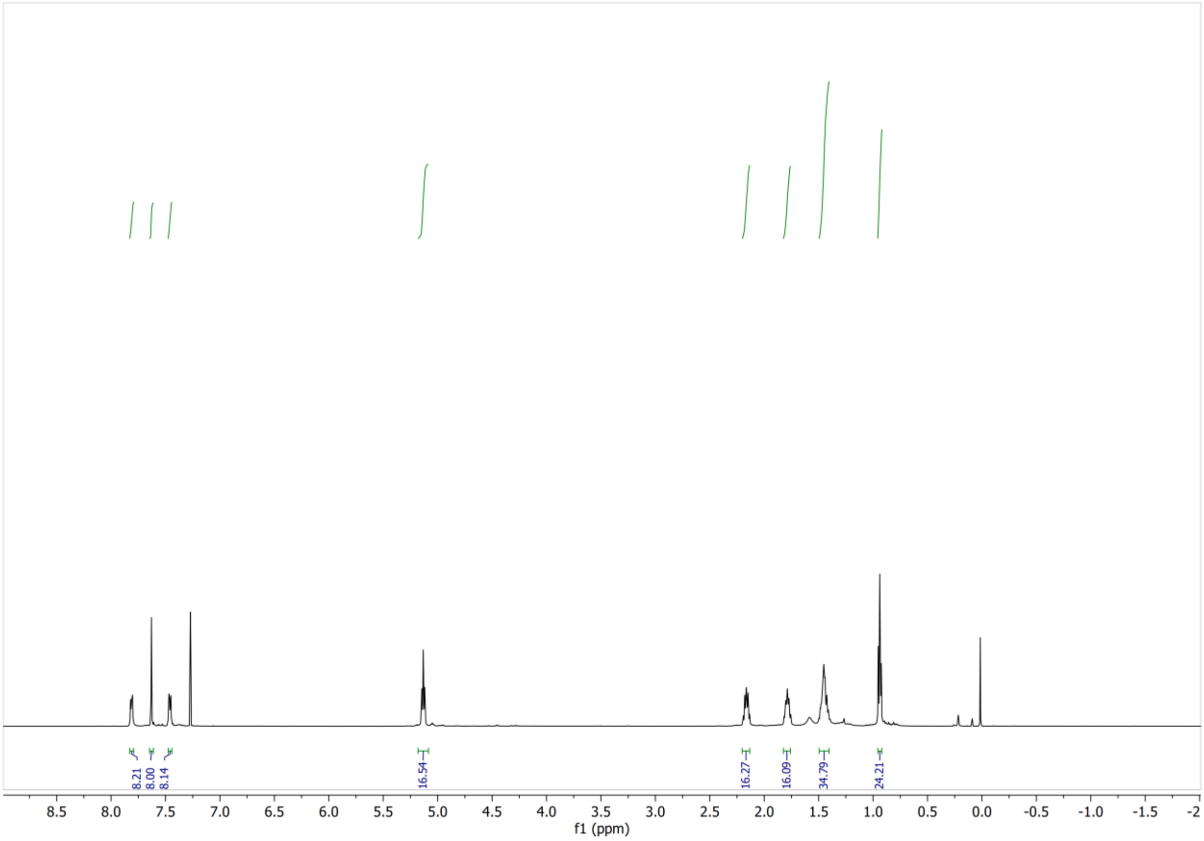

Figure S6. ^{1}H NMR spectrum of **5** in $CDCl_3$.

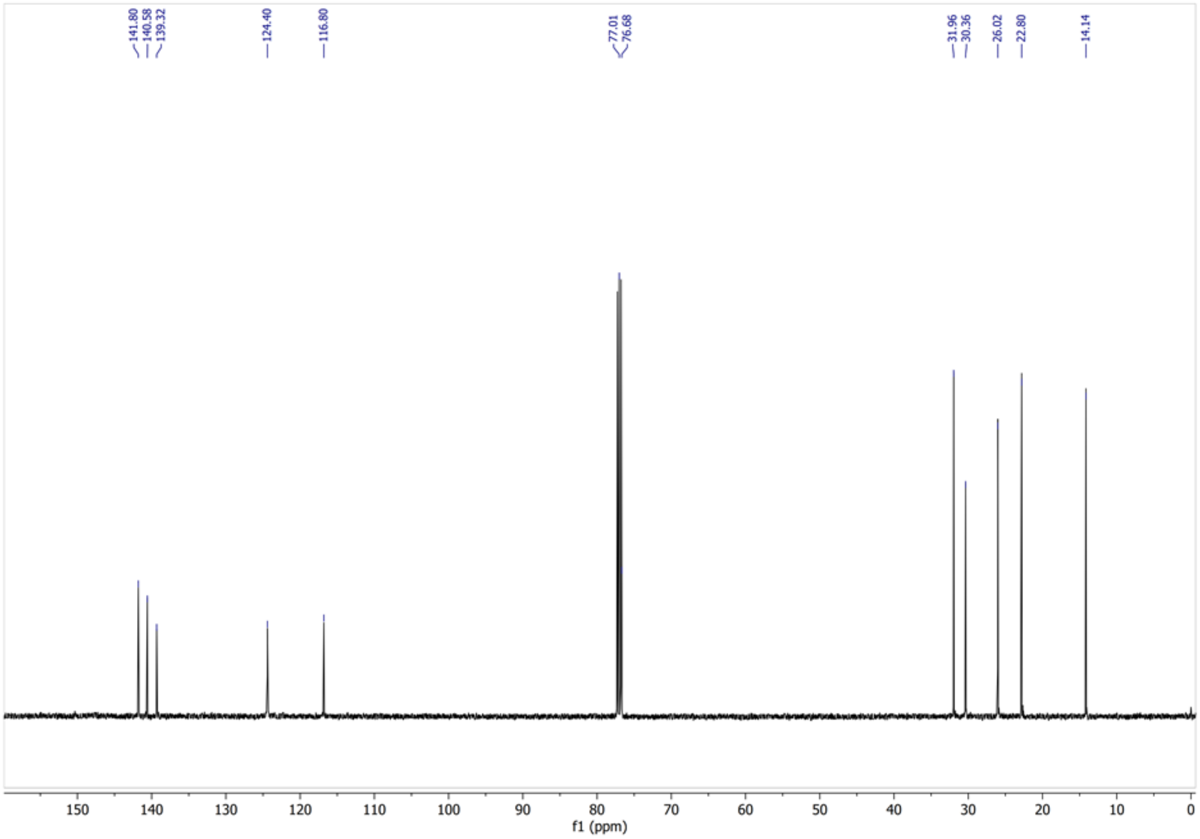

Figure S7. ^{13}C NMR spectrum of **4** in $CDCl_3$.

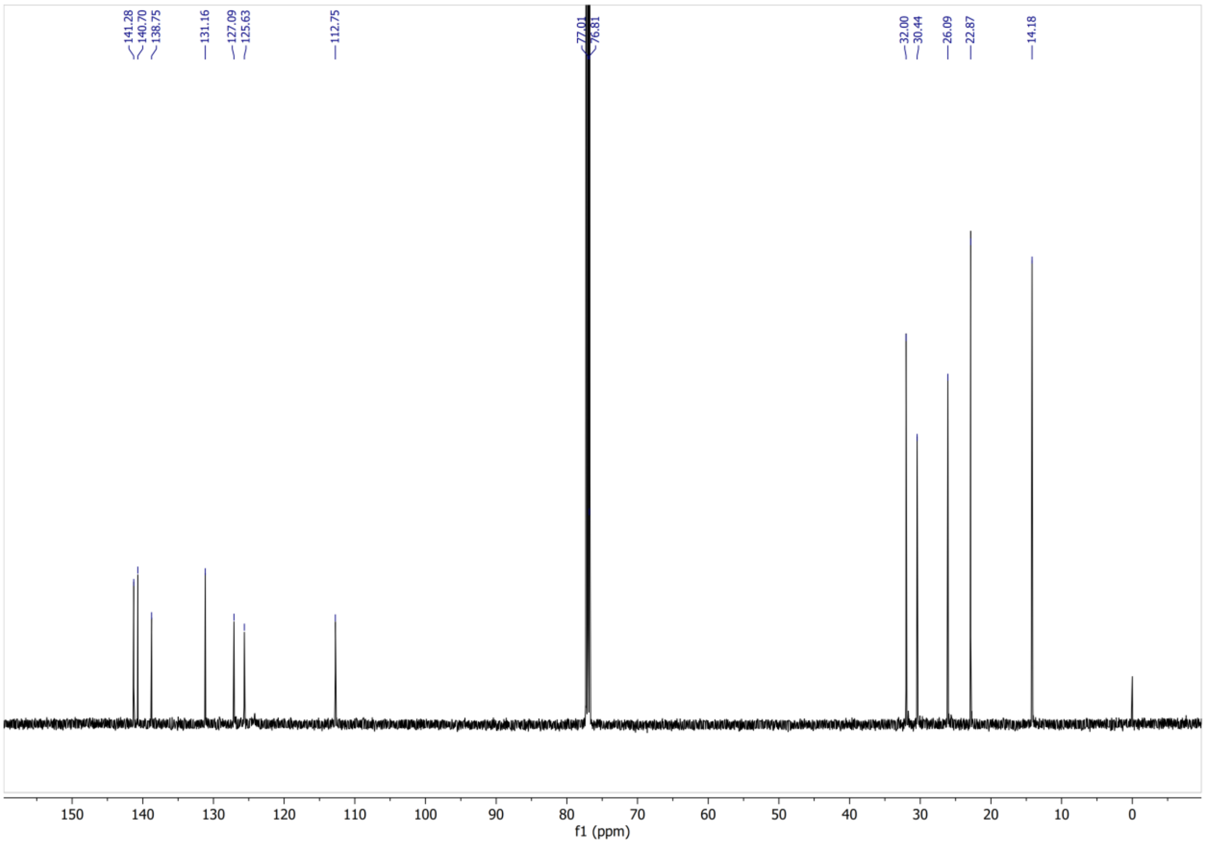

Figure S8. ^{13}C NMR spectrum of **5** in $CDCl_3$.

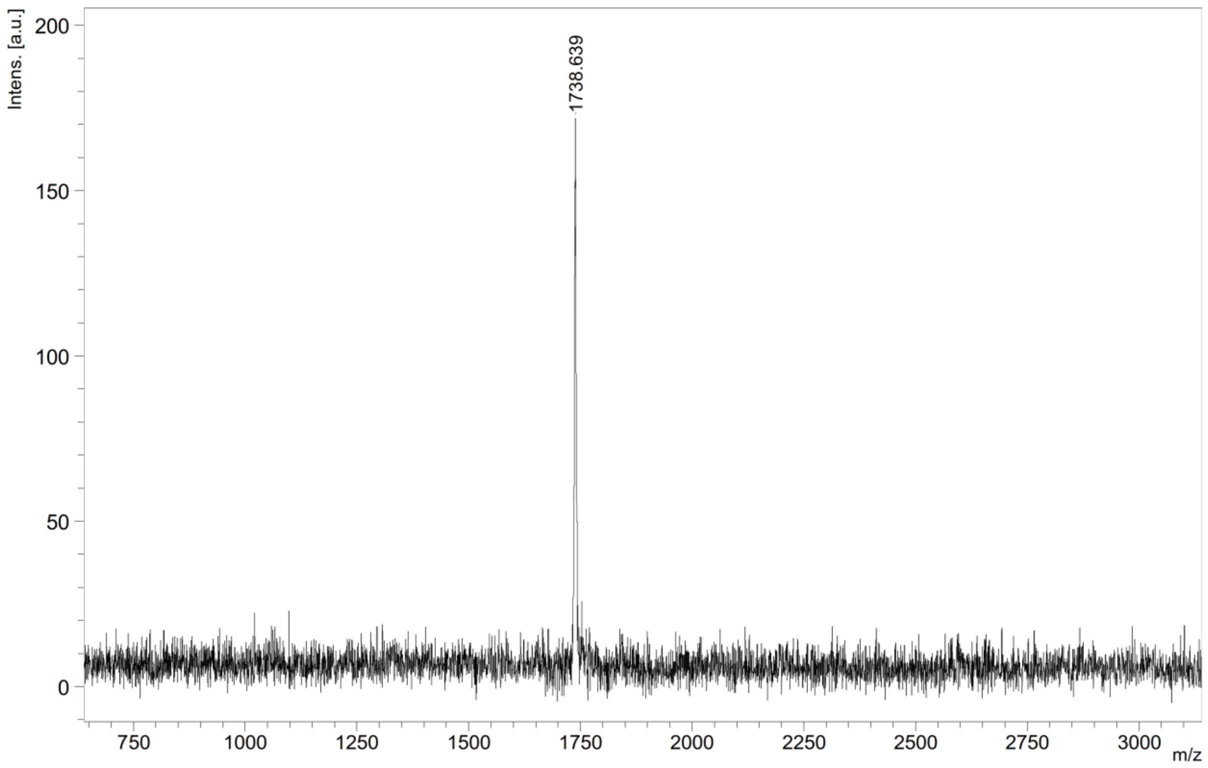

Figure S9. MALDI-TOF mass spectrum of **4**.

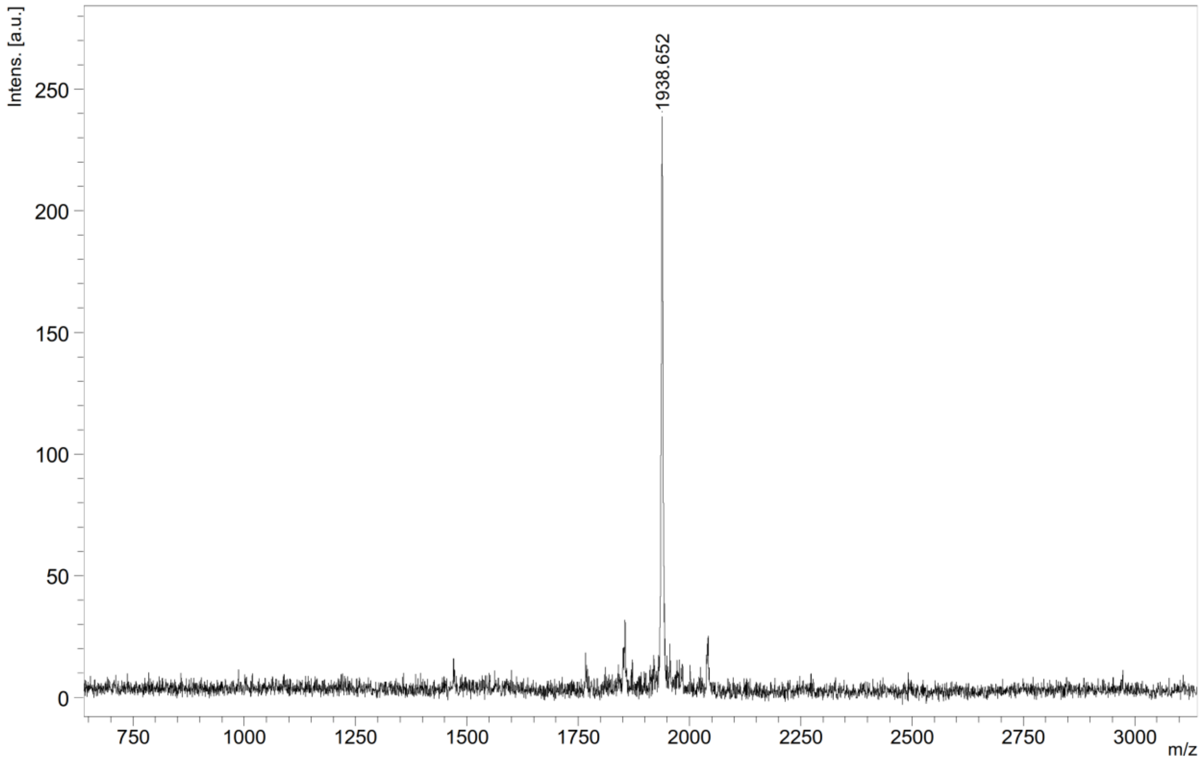

Figure S10. MALDI-TOF mass spectrum of **5**.

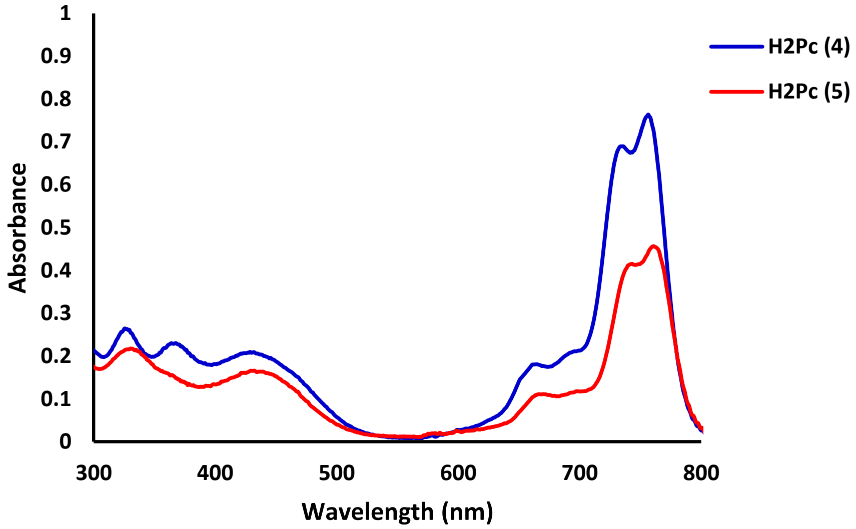

Figure S11. UV-Vis spectra of **4** and **5** in THF. (Concentration: 4 × 10^{-6} M).
